# AFLP and MS-AFLP Analysis of the Variation within Saffron Crocus (*Crocus sativus* L.) Germplasm

**DOI:** 10.1371/journal.pone.0123434

**Published:** 2015-04-17

**Authors:** Matteo Busconi, Licia Colli, Rosa Ana Sánchez, Marcela Santaella, Marcelino De-Los-Mozos Pascual, Omar Santana, Marta Roldán, José-Antonio Fernández

**Affiliations:** 1 Faculty of Agricultural, Food and Environmental Sciences, Università Cattolica del Sacro Cuore, Piacenza, Italy; 2 BioDNA, Centro di Ricerca sulla biodiversità e sul DNA antico, Università Cattolica del Sacro Cuore, Piacenza, Italy; 3 IDR-Biotechnology, Universidad de Castilla—La Mancha, Albacete, Spain; 4 Centro de Investigación Agraria de Albaladejito, Consejería de Agricultura de Castilla–La Mancha, Cuenca, Spain; Agriculture and Agri-Food Canada, CANADA

## Abstract

The presence and extent of genetic variation in saffron crocus are still debated, as testified by several contradictory articles providing contrasting results about the monomorphism or less of the species. Remarkably, phenotypic variations have been frequently observed in the field, such variations are usually unstable and can change from one growing season to another. Considering that gene expression can be influenced both by genetic and epigenetic changes, epigenetics could be a plausible cause of the alternative phenotypes. In order to obtain new insights into this issue, we carried out a molecular marker analysis of 112 accessions from the World Saffron and Crocus Collection. The accessions were grown for at least three years in the same open field conditions. The same samples were analysed using Amplified Fragment Length Polymorphism (AFLP) and Methyl Sensitive AFLP in order to search for variation at the genetic (DNA sequence) and epigenetic (cytosine methylation) level. While the genetic variability was low (4.23% polymorphic peaks and twelve (12) effective different genotypes), the methyl sensitive analysis showed the presence of high epigenetic variability (33.57% polymorphic peaks and twenty eight (28) different effective epigenotypes). The pattern obtained by Factorial Correspondence Analysis of AFLP and, in particular, of MS-AFLP data was consistent with the geographical provenance of the accessions. Very interestingly, by focusing on Spanish accessions, it was observed that the distribution of the accessions in the Factorial Correspondence Analysis is not random but tends to reflect the geographical origin. Two clearly defined clusters grouping accessions from the West (Toledo and Ciudad Real) and accessions from the East (Cuenca and Teruel) were clearly recognised.

## Introduction

Saffron is the highest priced High Value Agricultural Product (HVAP) in the world; its price can reach 20,000 €/kg retail. The saffron crocus (*Crocus sativus* Linnaeus 1753) is a perennial and genetically sterile plant that is only vegetatively propagated via its corms, which undergo a period of dormancy. This plant has been traditionally cultivated for its red stigmatic three-branched styles that contain both a highly desirable “golden condiment” and a number of secondary chemical derivatives, which have been used in medicine for a number of health properties. Unfortunately, the sterility in saffron crocus limits the application of conventional breeding approaches for further improvement [1].

*C*. *sativus* is a sterile triploid (2n = 3x = 24), initially assumed to be autotriploid, although a growing amount of evidence supports alloploidy as the most probable mechanism of origin. *C*. *cartwrightianus* seems to be a probable parental, providing two out of the three genomes, but the other parental species remains unclear [1]. In order to establish biodiversity conservation strategies and crop improvement programmes, it is important to ascertain the levels of genetic diversity within the species *C*. *sativus*. The crop multiplies year by year by means of corms. Corm multiplication does not generate genome variation, however, with the exception of a few somatic mutations that are not easily detectable in a triploid species. Thus, if a single polyploidisation plus domestication event had occurred, it can be hypothesised that all saffron crocus clones are similar to each other. At present, however, the presence and extent of genetic variation in the species is still debated, as testified by several contradictory articles providing contrasting results about the monomorphism of the species.

In a unique, large-scale project, Agayev et al. [2] showed a rapid and stable response of *C*. *sativus* to clonal selection. The best performing plants were identified and selected from a large number (many thousands) of plants from various Iranian populations, being able to create true “cultivars”. The efficacy of clonal selection suggested genetic diversity in the species.

The karyotype of *C*. *sativus* has been studied by a number of authors, and, while contemporary accessions from different countries usually reveal a common karyotype without major differences, other karyotypes have been described in the former literature [1, 3].

Interestingly, Rubio-Moraga et al. [4] stated that saffron crocus is a monomorphic species, deriving their conclusion from the fact that no clear polymorphic signals were found when analysing accessions from various countries with different molecular markers (RAPD, ISSR, SSR). Divergently, other authors identified low genetic differences by using EST-derived SSR [5, 6]. Single Nucleotide Polymorphisms (SNPs) in different accessions, which are often heterozygous, have been detected by direct sequencing (Fernández, unpublished data). Siracusa et al. [7] detected genetic differentiation between samples from different geographical areas (Europe and Asia) using AFLP markers, although no evidence of any significant phenotypic variation of samples of different geographical provenience was noted. Interestingly, the same authors highlighted the presence of intra-accession variability by analysing four plants for each accession. These works clearly demonstrate that the situation is still unclear, having evidenced alternatively no-variability, low variability, and variability in *C*. *sativus*. Remarkably, phenotypic variations have been frequently observed in the field by researchers and saffron producers: plants with a different number of stigmas or with different aspect of tepals. Interestingly, such phenotypic variations are occasionally unstable and can change from one growing season to another [1].

The loss of land surface dedicated to saffron crop in many areas of Europe has led to genetic erosion. Hence, the creation of a germplasm bank for this species, including wild relatives to broaden the gene pool available for genetic improvement, represented a great achievement. Since 2007, the European Commission AGRI GEN RES 018 Action (CROCUSBANK Project) has permitted the creation of the World Saffron and Crocus Collection (WSCC), located in the Bank of Plant Germplasm of Cuenca (BGV-CU), Spain, and which contains a reasonably good representation of saffron crocus biodiversity, although there is a high abundance of Spanish accessions (see www.crocusbank.org for details). Nonetheless, the germplasm bank includes more than 443 accessions representing 54 different *Crocus* taxa, with 197 *C*. *sativus* accessions from 15 countries that represent the largest world-scale collection of saffron crocus genetic resources. According to the clonal selection results of Agayev et al. [2], the preliminary characterisation of the WSCC highlighted the presence of phenotype variability between and within accessions for different morphologic and agronomic traits [8, 9]. That raises the question about the origin of the detected phenotypic variability.

Plants are sessile organisms that have to face a wide variety of environmental changes and challenges, consequently the availability of fast inducible perception and adaptation strategies is extremely important for their survival. Many plant genes are known to be regulated by biotic and abiotic stresses. In recent years, there has been evidence suggesting a key role of epigenetic mechanisms (plant small RNAs, DNA methylation and histone modifications) in regulating plant responses to stresses [10, 11, 12, 13, 14]. Epigenetics changes are meiotically or mitotically heritable modifications in gene function that are not due to changes in DNA sequence [15](Bonasio et al., 2010). At the molecular level, DNA methylation of cytosine with the conversion to 5-methylcytosine is one of the most widespread epigenetic modifications. DNA methylation of cytosines in plants can occur at different genomic sites: CG, CHG and CHH, where H can be A, T or C. As epigenetic marks, DNA methylation may alter strongly the structure of chromatine, deeply influencing eukaryotic gene expression and the development and environmental responses of plants likewise. Recent works have shown that epigenetics plays a key role in the proper regulation of self-incompatibility, sex determination, shoot regeneration and genomic imprinting [16]. In recent years, an increasing number of studies have evidenced that some epigenetic marks can persist through DNA replication and can be stably transmitted to the following generations [17]. Stable, heritable epialleles (whose expression depends on their epigenetic status) causing heritable phenotypic variations are known in plants. So, scientists have begun to argue about the possible role for epigenetics in crop breeding, transgene regulation and epialleles creation [17]. Considering that gene expression can be influenced by both genetic and epigenetic changes, epigenetics could be a plausible cause of the alternative phenotypes observed in saffron crocus.

In order to obtain new insights, we performed a molecular marker analysis of the WSCC by using AFLP (Amplified Fragment Length Polymorphism) markers that, according to Siracusa et al. [7], seem to work well in characterising the presence of *C*. *sativus* genetic variability. In addition, the same samples have also been analysed using the methyl-sensitive variant of the AFLP methodology, called MS-AFLP (Methyl Sensitive Amplified Fragment Length Polymorphism) or MSAP [10, 14]. This second technique has been adopted to test the presence of epigenetic polymorphisms in the methylation state of the saffron crocus genome.

## Materials and Methods

### Plant material

Seventy-three (73) accessions from different Spanish areas and 39 accessions from different European and non-European countries were selected from the WSCC (S1 Table). The term accession, as intended for inclusion in the germplasm collection, indicates a sample of corms received at the BGVCU, which was integrated, propagated and managed in the collection. Each sample was labelled with a unique identifier which also included the number representing the original accession within the germplasm collection (BCU number). All passport data and related information of each individual accession (e.g. origin, type of material, etc.) were recorded in the Documentation System of the BGV-CU. All accessions were grown in the same field at the experimental farm of the BGV-CU (Spain) for at least three years under common cultivation practices and open field conditions. Previous analyses, carried out at the time of the establishment of the germplasm, showed that the soil composition was not significantly different in the various part of the field. In order to investigate the intra-accession genetic variability, we selected 19 out of the 73 Spanish accessions (the geographically and ecologically most representative) and analysed four to five plants of each accession. To investigate the epigenetic variability for all 73 accessions, three to five plants of each accession were analysed. Globally, 188 single samples were used in the search for genetic variations and 385 single samples were considered in the search for epigenetic variations.

### DNA extraction

Green leaves were collected at the end of the vegetative period in April 2012 during a single day and stored at -80°C. Leaves were subsequently ground and stored at -20°C until DNA extraction. Then, 200 mg of tissue powder was weighed and DNA extractions were carried out using the commercial kit DNeasy Plant Mini Kit (QIAGEN) according to the manufacturer’s instructions. The purified DNAs were visualised by means of 1% agarose gel electrophoresis and quantified by a NanoDrop 2000c UV-Vis Spectrophotometer (Thermo scientific). Considering the estimated large size of the *C*. *sativus* genome (circa 10 Gb), 250 ng of total DNA was used in the subsequent analyses.

### AFLP and MS-AFLP analyses

AFLP reactions were performed as reported in Siracusa et al.[7]. The digestion of 250 ng of genomic DNA was carried out using the standard restriction enzymes *Eco*RI and *Mse*I. All of the information about the primers for pre-selective and selective amplifications for both AFLP and MS-AFLP are reported in S2 Table. Pre-selective PCRs were carried out using E01 and alternatively M01 or M02 primers. Twelve different primer combinations were used for the selective amplifications: E38/M62; E32/M61; E32/M62; E36/M49; E38/M47; E36/M48; E32/M42; E38/M33; E32/M42; E35/M33; E35/M42.

MS-AFLP analyses were performed as reported by Marconi et al. [14]. The classic MS-AFLP approach involves the use of the isoschizomers *Msp*I and *Hpa*II in order to fully investigate the variation in the methylation state of the restriction site cytosines. Both enzymes cut the DNA if the restriction site is not methylated, but they cut in a different way in the presence of cytosine methylation. In this experiment, considering the high number of individuals (112 accessions, 385 individuals), the restrictions were carried out using only *Hpa*II as the methyl-sensitive enzyme. The restriction enzymes used were *Eco*RI and *Hpa*II. Pre-selective PCRs were carried out using E01 and HM0 primers. Three different selective primer combinations were used: E38/HM2; E32/HM3; E37/HM2. PCR amplifications were performed as reported in the literature [14].

The selective *Eco*RI primers were labelled with fluorescent dyes, and the amplified products from selective amplifications were visualised in an ABI Prism 3130xl Genetic Analyser (Life Technologies) and analysed using the GeneMapper Analysis software (Life Technologies). The electrophoretic patterns were visually inspected in the search for polymorphisms and the presence or absence of peaks was scored and compiled in a binary matrix.

### Statistical analysis of data

The software GenoType and GenoDive [18], specifically designed for studies on polyploid organisms with clonal reproduction, were used to (i) identify different genotypes: the threshold value for the discrimination between genotypes—epigenotypes was set to 1 mismatch for AFLP data and to 2 mismatches for MS-AFLP data, after calculating the mean number of mismatches between genotypes within accessions under an “infinite allele” model (0.72 and 1.64 for AFLP and MSAP, respectively); (ii) calculate clonal diversity indices: number of genotypes—epigenotypes, effective number of genotypes—epigenotypes and genetic diversity [19] corrected for sample size; and (iii) perform an Analysis of the Molecular Variance (AMOVA) [20] at the accession level, in order to evaluate the significance of genetic differentiation between accessions.

The relationships between individual multilocus genotypes at the population level were evaluated by a multivariate Factorial Correspondence Analysis approach (FCA) [21], using the software GENETIX 4.05.2 [22]. To infer population structure, an unsupervised Bayesian clustering was performed with the software STRUCTURE version 2.3.4 [23]: five independent replicates were performed for K values from 1 to 20 under the admixture model with correlated allele frequencies. The analysis was run following the recommendations given both by Structure software manual and by Falush et al. [23] concerning dominant markers and polyploid genotypes, and by adopting the following parameter set: 400000 Monte Carlo Markov Chains iterations following a 100000 burnin period, with no prior information on the population of origin (USEPOPINFO = 0) and accounting for the presence of recessive alleles (RECESSIVEALLELES = 1). According to a ‘recursive partitioning’ approach [24], the MSAP dataset was further split into two subsets corresponding to the major clusters identified at K = 2; these subsets were independently analysed with STRUCTURE software (five independent runs for K values from 1 to 10 with the same parameter settings detailed above). For all datasets (AFLP, MS-AFLPs and ‘recursive partitioning’ datasets), the best fitting K value was identified with the Evanno et al. [25] ΔK method. STRUCTURE results were graphically represented using Distruct ver. 1.1 [26].

## Results

### AFLP analysis

The DNA samples used for the analyses were of good quality with no evident smear on a 1% agarose gel. To avoid the possible influence of DNA extraction methods on the final results, all DNA was extracted using the same commercial kit as reported in previous works [27].

To ensure the reliability and reproducibility of the AFLP protocol, the analyses were replicated three times weekly for three consecutive weeks on a subset of accessions. At the end of these trials, the cross-checking of results showed a reproducibility of the banding pattern greater than 99%. After the reproducibility test, the method was applied to the whole sample set. All 804 peaks were visually scored from 12 primer combinations and 34 clear polymorphisms were identified, corresponding to 4.23% of the total (Table 1). The visual inspection made it possible to eliminate false positives, such as: a) peaks just above or below the threshold set; b) fluorescence blobs; and c) peaks too close together to be correctly resolved by the automated analysis. The percentage of polymorphic peaks for each primer combination ranged between 0 and 11.53%. Five (5) primer combinations produced only monomorphic peaks and just one combination (E35/M42) produced a percentage of polymorphisms greater than 10%. The 34 polymorphisms were almost equally divided into private (16 signals characteristics of individual samples) and shared (18 signals characteristics of more than one sample). In order to confirm the polymorphisms, the polymorphic samples were re-analysed: 1) by repeating the PCRs on the same restriction-ligation to see if the polymorphic signal was a PCR artefact; and 2) by repeating the restriction-ligation and all subsequent steps to confirm the AFLP profile obtained. The polymorphisms were always confirmed.

**Table 1:** Polymorphisms highlighted by the different classes of molecular markers.

Reaction	Primer combination	Polymorphic/total^a^	% polymorphism	Private/Shared polymorphisms^b^
**AFLP**	**E38/M47**	0/52	0	0
	**E32/M61**	1/43	2.32	0 P / 1 S
	**E38/M62**	0/72	0	0
	**E38/M33**	4/55	7.27	3 P / 1 S
	**E36/M42**	3/45	6.67	1P / 2 S
	**E35/M33**	2/64	3.125	1 P / 1 S
	**E35/M50**	7/85	8.23	2 P / 5 S
	**E36/M48**	8/90	8.89	2 P / 6 S
	**E36/M49**	0/66	0	0
	**E32/M42**	0/82	0	0
	**E35/M42**	9/78	11.53	7 P / 2 S
	**E32/M62**	0/72	0	0
	**Total**	34/804	4.23	16 P /18 S
**MS-AFLP**	**E32/HM3**	18/55	32.72	4 P / 14 S
	**E38/HM2**	11/42	26.19	1 P / 10 S
	**E37/HM1**	18/43	41.86	3 P / 15 S
	**total**	47/140	33.57	8 P / 39 S

Levels of polymorphism highlighted by the different primer combinations: number of polymorphic loci, percentage of polymorphism and ratio between private and shared polymorphisms.

Legend: a) ratio between polymorphic/monomorphic fragments scored; b) number of private and shared polymorphisms evidenced by each primer combination; private variants are present in single individuals while shared alleles are present in more than a single individual.

### AFLP statistical analysis

In the 188 analysed individuals, 12 different genotypes were detected (S3 Table): two genotypes were recognised as different when the pairwise comparison of their profiles showed more than a single mismatch. Along with a prevailing genotype (A1), 11 less frequent genotypes were found. Being characterised by the almost exclusive occurrence of genotype A1, Spanish, French and Greek accessions showed a uniform genetic make-up. The presence of small intra-accession variability was detected in some of the Spanish accessions, in particular inside BCU001610 (from Cuenca) and BCU001613 (from Albacete). These variations are sufficient to define the presence of alternative genotypes. The most variable set was represented by Iranian accessions, among which 5 different genotypes were found in 9 accessions. Genotype A1 was found to be spread in different production areas, while the other genotypes were restricted to single locations.

The Analysis of Molecular Variance (AMOVA) showed that only 3.2% of diversity existed at the between-accession level. Maximum diversity has been observed within populations (96.8%).

**Fig 1:**
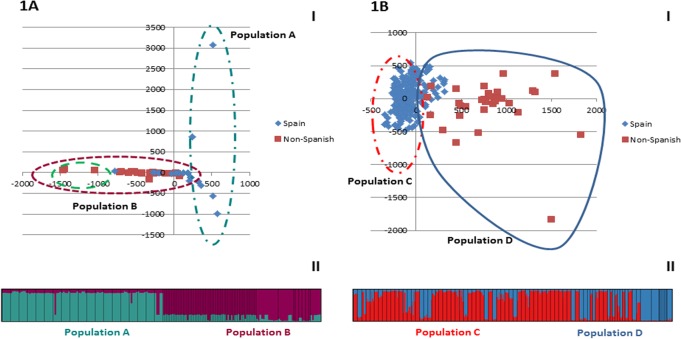
Factorial Correspondence Analysis evidencing the relationships among AFLP genotypes and MS-AFLP epigenotypes of different saffron crocus accessions. 1A) Factorial Correspondence Analysis showing multivariate relationships among AFLP genotypes of different accessions on the axes corresponding to first (x axis, 9.72% inertia) vs. second (y axis, 6.75% of inertia) main factors (1A I). The two populations Pop A and Pop B correspond to the main clusters identified by STRUCTURE analysis at K = 2 (1A II). Population A included just the S accessions and three different genotypes, while population B included both S and NS accessions and 9 different genotypes. Inside population B, samples included in the dashed line refer to accessions from Iran, India, Afghanistan and Turkey. 1B) Factorial Correspondence Analysis showing multivariate relationships among MS-AFLP epigenotypes of different accessions on the axes corresponding to first (x axis, 12.88% of inertia) vs. second (y axis, 10.15% of inertia) main factors (1B I). The two populations Pop C and Pop D correspond to the main clusters identified by STRUCTURE analysis at K = 2 (1B II). Population C included just Spanish accessions and 6 effective epigenotypes, while population D included both S and NS accessions and 22 effective epigenotypes.

The first two factors of the Factorial Correspondence Analysis (FCA) explained 16.47% of total inertia (Table 2). In particular, despite the overall reduced variability at the genomic level, the distribution of the points representing single accessions along the first factor (x axis in Fig 1) seems to correspond to the geographical provenance: most of the Spanish accessions (S) cluster on the right, with only a few points overlapping with Non-Spanish (NS) samples in the central part of the graph (Fig 1A I). Iranian accessions, instead, are located on the left of the plot and do not overlap with any other group. These results were also supported by *Crocus* diversity indices (S3 Table). In fact, excluding French and Greek accessions that have the same dominant genotype as Spanish ones (A1), the samples from other areas (Iran, Turkey, Italy and so on) have slightly different genotypes. Among S accessions, no relationship between geographic origin (different areas of Spain) and genotype can be defined, because the genotype was almost always the same (A1). While in Fig 1 the evident distinction is between S and NS origin, the same representation based on the effective genotypes is also provided (S1 Fig).

**Table 2:** Results of the FCA analysis for AFLP and MS-AFLP markers.

	AFLP Markers				MS-AFLP markers			
Factors	1	2	3	4	1	2	3	4
**%**	9.72	6.75	6.45	5.88	12.88	10.15	8.62	7.96
**Cum %**	9.72	16.47	22.92	**28.8**	12.88	23.03	31.64	**39.61**

Percentage of inertia explained by the first 4 factors of the Factorial Correspondence Analysis for AFLP and MS-AFLP markers.

According to the results of STRUCTURE software analysis, the best-fitting reconstruction of population structure was found at K = 2, as highlighted by the maximum ΔK score of the Evanno et al. [24] test (Fig 1A II). At K = 2, the saffron crocus specimens are split into two clusters (named population A and B in Fig 1A) corresponding to the major groups already highlighted by FCA analysis: all of the accessions grouped into population A were S and possessed genotype A1, with only two exceptions carrying genotypes A2 and A3 (S1 Fig), while population B included accessions with different provenance and genotypes.

### MS-AFLP analysis

The protocol adopted for MS-AFLP analyses was able to provide high reproducibility (higher than 98%) in independent reactions (data not shown). Three (3) primer combinations allowed a high level of polymorphism to be detected: 47 peaks out of the 140 scored (33.57%) were polymorphic (Table 1). The percentage of polymorphisms detected by each of the three combinations ranged between 26 and 42%. The level of MS-AFLP polymorphism was clearly higher than the level of AFLP polymorphism (33.57% against 4.23%). The number of shared polymorphisms (ca. 83% of the total) was greater than the number of private ones. Additionally, the analysis of MS-AFLP profiles revealed the presence of intra-accession variability at the epigenetic level. Some polymorphisms were characteristic of single accessions (all the individual plants from those accessions showing the signal), while others were shared by more accessions. The profiles were confirmed by repeating independent analyses on the samples with polymorphic peaks.

### MS-AFLP statistical analysis

The 47 polymorphic loci gave rise to 28 effective epigenotypes (S3 Table). To this end, two epigenotypes were considered different when having more than two mismatches. If a single mismatch was considered the threshold, the number of different epigenotypes would have increased to 70. For AFLP and MS-AFLPs, Spanish accessions were characterised by the presence of a dominant epigenotype together with a set of other epigenotypes occurring at a lower frequency, while the accessions from other geographic areas were characterised by very divergent epigenotypes. Globally, 10 effective epigenotypes were identified among S accessions, while the remaining 18 were found among NS samples. While at the genetic level, French and Greek accessions were characterised by a uniform genetic profile, at the epigenetic level, they showed several polymorphisms and 12 different epigenotypes.

Analysis of Molecular Variance (AMOVA) showed that only 2.7% diversity existed at the accession level. Maximum diversity was observed within populations (97.3%).

The first four factors of the Factorial Correspondence Analysis (FCA) on MS-AFLP markers explained 39.61% of the cumulative inertia (Table 2). When plotted on a graph, the first two factors highlighted a clear difference between the epigenetics of S and NS accessions (Fig 1B I): similar to the FCA plot of AFLP data (Fig 1A I), the pattern was consistent with the geographical provenance of the accessions for MS-AFLP epigenotypes as well, while no environmental effects were revealed. In fact, the distribution of single accessions on the graph did not highlight any clustering consistent with the time elapsed since the beginning of cultivation in uniform environmental conditions at the germplasm bank (S2 Fig). Having a more uniform epigenetic make-up, S accessions are grouped together in the upper left part of the graph, while NS accessions coming from cultivation areas characterised by extremely different climatic conditions were more scattered, with a very limited overlap between the two groups. In Fig 1B, the distinction is between S and NS origin, while the same representation is provided in S3 and S4 Figs, but this is based on: a) the different epigenotypes and b) the clustering of accessions according to STRUCTURE software results, respectively.

The reconstruction of population structure with STRUCTURE software showed a Δk peak at K = 2: but the clustering does not correspond to that highlighted by the FCA plot: in fact, the accessions assigned to the two separate clusters by STRUCTURE partly overlap in the FCA graph (Fig 1B II and S4 Fig). All of the accessions inside population C were S and represented by 6 effective epigenotypes (M1, M2, M3, M5, M6, and M10), while population D comprised accessions with different origins and 23 effective epigenotypes (epigenotype M1 is present in both clusters). Following the recursive partitioning approach, the two main groups defined by STRUCTURE (pop C and pop D) were further and independently analysed with the same software. Population D was further subdivided in two sub-populations roughly corresponding to Spanish and Non-Spanish accessions. On the contrary, the Spanish accessions inside population C were clustered in 7 sub-populations (S4 Fig).

**Fig 2:**
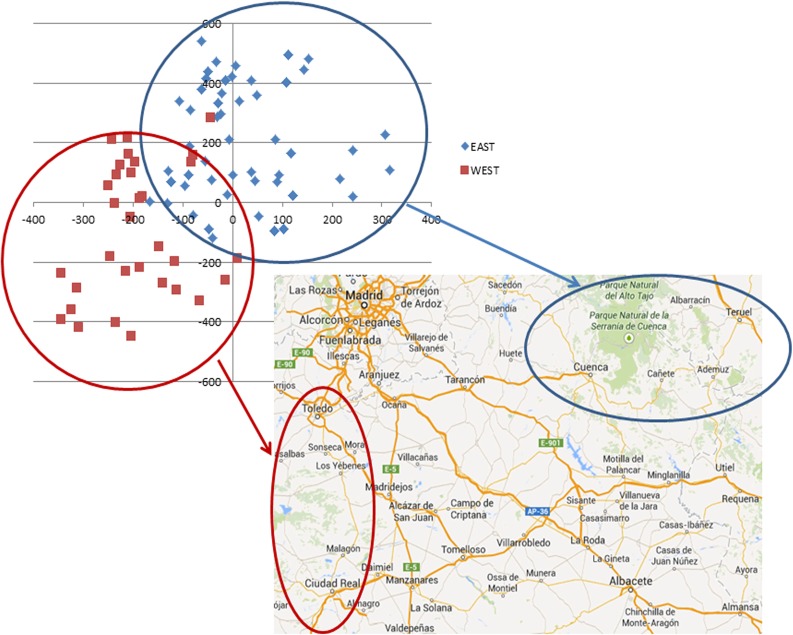
FCA analysis based on the MS-AFLP epigenotypes of Spanish accessions. Factorial Correspondence Analysis showing multivariate relationships among MS-AFLP epigenotypes of different saffron crocus accessions of Spanish provenance on the axes corresponding to first (x axis, 12.88% of inertia) vs. second (y axis, 10.15% of inertia) main factors. Only the points corresponding to accessions of Spanish origin have been plotted. Accessions from the WEST (Toledo and Ciudad Real) and from the EAST (Cuenca and Teruel) tended to cluster separately with just few exceptions. Climatic conditions between the two areas are very different and this may be reflected in the epigenetic composition.

While no relationship between geographical region of origin and genotypes could be defined for S accessions with AFLPs, a different situation emerged with MS-AFLPs (Fig 2). Almost all of the S accessions (67 out of 73) were from the administrative regions of *Castilla-La Mancha* (provinces of Toledo, Ciudad Real, Albacete, and Cuenca) and *Aragón* (province of Teruel). By focusing on these samples, it was possible to observe that their distribution in the FCA scatterplot is somewhat influenced by the geographical origin. Accessions coming respectively from the EAST (Cuenca and Teruel) and from the WEST (Toledo and Ciudad Real, light grey) tended to cluster together in two separate groups with only a few exceptions, while accessions from Albacete (South-East part of Castilla–La Mancha), bordering the provinces of Ciudad Real and Cuenca, tended to be in the middle and partially superimposed to the two groups (S5 Fig).

## Discussion

Saffron crocus is not strictly monomorphic but the presence of little genetic variability has been revealed by analysing several accessions preserved at the BGV-CU, originating mainly from Spain but also from other European and extra-European countries. These findings support and strengthen what has already been reported by Siracusa et al. [7] on a limited number of samples. Recently, Erol et al. [28] successfully used AFLP markers to analyse the relationships within the genus *Crocus* and here we confirmed that these markers are also useful for detecting genetic variability in the saffron crocus germplasm. The reproducibility of the AFLP protocol is crucial if the aim is to ascertain the presence or absence of genetic variability in a species which, very likely, has a clonal origin. For this reason, the evaluation of polymorphisms was carried out with the maximum stringency in order to avoid false positives as much as possible. The reproducibility of the protocol employed was higher than 99%, allowing the discrimination threshold to be set between the genotypes at a single mismatch. The presence of polymorphic signals shared between accessions of different provenance suggested an ancestral origin from a single corm. The polymorphisms present in more samples, independently of their origin, were probably based on mutations older than those causing the sample-specific polymorphisms. The finding of shared polymorphisms in European, African, and Asian accessions fits the hypothesis of a single origin followed by the frequent exchange of corms between different cultivation areas in the past. This view was also supported by the presence of a predominant genotype that was widespread across different production areas. All of this fits with the hypothesis of a clonal origin of the samples followed by the accumulation of random mutations. Among the different accessions, the highest number of different genotypes was observed within the accessions from Iran. Globally, the total amount of genetic differences was sufficient to cluster the samples into two main groups: the first included only Spanish accessions, while the second included accessions with several origins. This was probably a consequence of the fact that the majority of the accessions were of Spanish origin. Some of the Non-Spanish samples of population B were geographically closer to the Spanish ones of population A than the French and Greek accessions, while others were more distant. Interestingly, the samples of population B that are more distant from the Spanish ones were from India, Iran, Afghanistan and Turkey (see Fig 1A). This can be expected considering that those Asian countries have an ancient history of saffron crocus cultivation and that the plant was likely reintroduced in Europe from those countries during the middle ages by crusaders and Arabs. More recently, some differences between Iranian accessions have also been highlighted also by using SSR [29]. Taken together, this information allows speculation on the reasons why no variations were found in previous studies. In fact, as our results suggest, using accessions from different countries does not automatically enable the detection of variation, due to the presence of a main genotype (A1) being widespread in several accessions from different areas. Using multi-locus markers (AFLP) can improve the possibility of detecting variability with respect to single-locus markers like SSR, despite their high polymorphism. The detection system based on AFLPs can improve the survey of variability and, concerning this, automated systems are better than standard gel-based systems. Finally, the variations that we detected are mainly based on single nucleotide polymorphisms at the level of the restriction site, as random single base mutations are more likely to appear with respect to bigger changes (insertion or deletion of more nucleotides, changing in the annealing site of primers) that are detectable by other marker systems like SSRs or RAPDs. Therefore, contrary to that which was reported in some articles [4, 30]), here we report that it was possible to score the presence of some genetic variation. The polymorphisms detected were probably the consequence of spontaneous mutations that randomly arose in different samples during the 3–5 thousand years elapsed since the domestication of *C*. *sativus*. The presence of genetic variability, even if small, can justify efforts to preserve different accessions in a germplasm collection. Supporting this, Agayev et al. [2] reported some first optimistic results of a unique, large-scale research project that focused on the problem of clonal selection of saffron crocus. Based on the number of flowers and number of large corms, which are the two most economically important attributes of the plant, the clones were classified by the authors as extraordinary, superior, ordinary, inferior and declining clones.

Whether the small genetic variability detected can influence the phenotype still has to be demonstrated. So far, no correlation between genetic variability and phenotype was detected [7], but further research, supported by the application of next generation sequencing approaches, is required to ascertain this issue.

Nevertheless, high phenotypic plasticity is present in the field and it was often reported that the changes are unstable, shifting from one growing season to the other. Phenotypic plasticity refers to the ability of an organism to produce different phenotypes under different environmental conditions. Epigenetic modification provides a plausible mechanism for the putative link between environmental variations and changes in gene expression, and the consensus is growing stronger on the idea that epigenetics can complement genetic variation as a source of phenotypic variation, at least in natural plant populations [31].

Epigenetic variations, contrary to genetic variations, are influenced by environmental conditions, so it is very likely that samples from cultivation areas under different climates can be characterised by different epigenomes. To reduce this effect, despite their geographical origin and after being received at the BGV-CU, the samples were grown under open field conditions in the same field for at least three years to obtain a better standardisation of the epigenomes. In fact, although epigenetic states are stable by definition, they can revert at certain frequencies [32]. Therefore, what we observed in this study may also be due to the combined effect of the original epigenetic state (before cultivation in the germplasm bank) and of the epigenetic changes induced by cultivation in the bank under uniform environmental conditions. This view is supported by the fact that, despite having been cultivated in the bank for at least 3 years, the accessions from different areas still have different epigenotypes. In fact, except for the widely spread M1, all other epigenotypes are private to single accessions. Indeed, according to some authors, those reversible changes, while having an epigenetic base, must just be considered part of the phenotypic plasticity while a true epigenetic mark should be stable and stably transmitted to the progeny through cellular divisions (meiosis and mitosis) for several cycles [33]. This phenomenon is known as Transgenerational Epigenetic Inheritance (TEI). The presence of a small level of intra-accession variability which was observed in this experiment could be a consequence of the phenotypic plasticity and may be reversible. It is not possible to exclude the hypothesis that such reversible polymorphisms could determine some of the reversible phenotypes observed in the field.

With respect to the MS-AFLP analysis, the epigenetic variability is very high and just a few primer combinations were sufficient to highlight the presence of a high number of polymorphisms. Not all of the polymorphisms were registered, only those that were more clearly readable; several polymorphic signals, which were not very clear, were not considered. Nevertheless, the number of polymorphisms detected just with three primer combinations was higher than that scored with twelve AFLP combinations. The polymorphism detected by MS enzymes can have both a genetic and epigenetic origin, so it is possible that a small part of the 33.57% of polymorphic signals, likely a percentage similar to AFLP, is based on genetic variation, even though its actual extent could be measured only by coupling both isoschizomers as in the classical MS-AFLP approach.

We can propose some hypothesis to explain the higher epigenetic variability (33.57% compared to 4.23% genetic variation) and the relevance of residual epigenetic variability inside saffron germplasm. The origin of the saffron crop is still uncertain but, very likely, not as old as other domesticated crop. Archaeological and historical evidence indicate that domestication of saffron crocus dates back to 2,500–1,500 years BC. Further, being a sterile triploid and lacking any form of sexual reproduction, saffron crocus multiplication and spread was achieved mostly through human mediated corm propagation. A recent study carried out in domesticated grapevine (*Vitis vinifera* L.) has evidenced that the highest percentage of existing genetic diversity likely derives from random spontaneous somatic mutations in vegetative propagated material rather than from sexually derived polymorphisms [34]. Contrary to this, while recognising the difference between grape and saffron and the oldest domestication of grape, it seems that the situation is different in saffron crocus where just rare spontaneous mutations can be detected. Consequently, not being able to rely on genetic variation, epigenetics represented the only way the crop had to adapt to the extremely different environments where its cultivation was spread during history. This can explain why such a high level of epigenetic variability has been found inside the species.

Epigenetics is involved in different aspects of plant development, mainly in gene regulation and transposable element (TE) control. TEs are mobile elements that can induce mutations and chromosome instability. TEs, especially the LTR retrotransposons, are very abundant in plant genome and a strong correlation exists in angiosperm species between TE content and genome size [35]. The size of saffron crocus genome has been estimated to be greater than 10 Gb [36]. As other big plant genomes, it consists mainly of repetitive DNA sequences as retrotransposon and satellite DNA, the latter forming long tandem arrays in large heterochromatic blocks at distinguished chromosomal positions [37]. Epigenetics marks affecting TEs are stable over time and extremely important in keeping TEs silenced by preventing their mobilisation [38]. We can hypothesise that the epigenetic differences that we still found in saffron crocus despite years of cultivation in the germplasm bank are due to stable epigenetic marks involved in TEs silencing and control, while the reversible epigenetic marks may already have changed as a consequence of the new climatic conditions.

In the definition of the effective number of epigenotypes, we considered 2 mismatches, mainly because the reproducibility of the protocol was higher than 98%; however, if we use the same parameters as for AFLP, the number of effective epigenotypes would increase significantly (70 with respect to 28). Contrary to that which was observed with AFLP, in this case, the Non-Spanish accessions were characterised by very different epigenotypes. For example, French and Greek accessions, having the same genotypes as the Spanish accessions, had their own epigenotypes (S3 Table). It must be said that the sampling of the accessions was carried out during a single day but in different hours: some accessions were sampled early while other late in the morning. We cannot exclude that this difference may have slightly affected the whole plant epigenome

Two main factors may have influenced the current epigenetic state of the accessions: the geographic origin and the lapse of cultivation in the same environmental conditions. Considering this, the results of the FCA analysis were alternatively represented according to the epigenotype (S3 Fig), to the time of cultivation in the germplasm bank (S2 Fig), and to the origin of the accessions (Fig 2). No particular relationship between time of cultivation and epigenetic state was observed. The situation was different when the provenance was considered: the epigenetic differentiation between Spanish and Non-Spanish accessions was larger than the overall genetic differentiation scored with AFLPs (Fig 1B and S3 Fig). Furthermore, while Spanish samples have a more uniform epigenetic state, Non-Spanish samples are widespread on the graph and clearly distinct from the Spanish ones. The analysis with STRUCTURE software revealed the presence of two main clusters: population C grouping only Spanish samples and population D grouping both Spanish and all of the Non-Spanish samples (Fig 1B and S4 Fig). The separate analysis of population D still showed the possibility of recognising Spanish from Non-Spanish. This second population mix of accessions with different origins may be the result of some standardisation of the germplasm because of its cultivation under the same conditions. The separate analysis of population C evidenced the presence of at least 7 main sub-populations but no clear relations between their epigenotypes and the geographic origin or time of cultivation were highlighted.

Interesting results were obtained when we decided to focus our attention on Spanish accessions (Fig 2 and S5 Fig). In fact, it was observed that the distribution of the accessions in the FCA analysis is not random but tend to reflect the geographic origin of the samples. Two clearly defined clusters, the one grouping the accessions from the West (Toledo and Ciudad Real) and the second grouping the accessions from the East (Cuenca and Teruel), can be easily recognised. Further, samples coming from the intermediate province of Albacete are spread in the middle between the other two clusters. A similar situation cannot be observed in relation to the time of cultivation in the BGV-CU. Climatic, soil and ecological conditions in general are highly different in the western and in the eastern parts of Central Spain and this can be reflected in the epigenetic profiles of the samples. This supports the fact that the different accessions tended to maintain an epigenetic state through the years that is, at least partially, the original one, making it possible to group together those accessions with closer geographic origins.

It appeared possible that, despite years of cultivation in the same climatic conditions, the different accessions still conserved some original epigenetic marks, likely derived from several years of cultivation in their countries of provenance. It is known that stable epigenetic states in plants can be propagated over many meiotic and mitotic replication cycles. Although a series of studies has suggested that the transmission of environmentally induced traits may occur, it is likely that some of the plant plastic responses to environmental challenges are not transmitted to the progeny, being reset between generations. It is still not clear what determines whether an epigenetic state is transmitted or reset [31]. The existence of naturally occurring epialleles, influencing specific phenotypes that are stably transmitted to the progeny through TEI, is known in plants. A prominent example of naturally occurring TEI in plants is the case reported by Paszkowski and Grossniklaus [31] of an epiallelic variant at the *Cycloidea* locus causing the radially symmetric, peloric phenotype of *Linaria* flowers; the involvement of DNA methylation in its formation and maintenance for hundreds of years in nature is well documented. Considering this, it was possible to hypothesise that, in the sterile species *C*. *sativus*, which propagates only through the mitotic formation of new corms, the epigenetic status can be highly stable and transmitted to the new plants. While some epigenetic modifications can persist over time once established, other modifications may be only temporary. The observed instability of some alternative phenotypes in *C*. *sativus* can be explained by considering a reversible epigenetic basis. Future studies following the transmission of the epigenetic state will be carried out by comparing the epigenome of the offspring of single corms through different generations. Recently we started a study based on the comparison of the epigenetic variability occurring within the same accessions in different years. For some of the accessions considered in this paper, we are comparing the MS-AFLP profile of leaves sampled at the end of April 2012, 2013, and 2014. Some preliminary results indicate that, although some small changes can be present, globally the epigenetic profiles of single accessions are highly superimposable across years. Such observations suggest a high stability of saffron crocus epigenome vegetatively propagated from mother to offspring corms in subsequent years (S6 Fig). This can open to the possibility of using epigenetics variation for breeding as already suggested in literature [17], but it will be first necessary to evaluate epigenetic stability, also at the level of single corms and their progeny, and to test the association of stable epigenetic marks to specific saffron crocus phenotypes.

As closing remarks, we can report that little genetic variability and high epigenetic variability are present inside the saffron crocus germplasm. We can hypothesise that the good results obtained by Agayev et al. [2] were also based on stable epigenetic marks transmitted to the offspring through TEI. Genetic variability in *C*. *sativus* is most likely a consequence of spontaneous mutations and all of the accessions shared a very similar genetic constitution. This offers new opportunities for basic research, and saffron may become an election crop for epigenetic studies, in order to investigate how a same genotype can adapt to different environmental conditions.

## Supporting Information

S1 Fig Factorial Correspondence Analysis based on the different genotypes as defined by GenoType and GenoDive software. Factorial Correspondence Analysis showing multivariate relationships among AFLP genotypes of different *C*. *sativus* accessions on the axes corresponding to first (x axis, 9.72% of inertia) vs. second (y axis, 6.75% of inertia) main factors. The different accessions are represented according to the AFLP genotype, as defined by the analysis with GenoType and GenoDive software.
(TIF)

S2 Fig Factorial Correspondence Analysis based on the year of the first sowing in the germplasm. Factorial Correspondence Analysis showing multivariate relationships among MS-AFLP epigenotypes of different *C*. *sativus* accessions on the axes corresponding to first (x axis, 12.88% of inertia) vs. second (y axis, 10.15% of inertia) main factors. The accessions are represented according to the year of the first sowing in the germplasm bank. S accessions were received along the entire time while NS accessions were only received in 2006, 2008 and 2010.
(TIF)

S3 Fig Factorial Correspondence Analysis based on the different epigenotypes as defined by GenoType and GenoDive software. Factorial Correspondence Analysis showing multivariate relationships among MS-AFLP epigenotypes of different *C*. *sativus* accessions on the axes corresponding to first (x axis, 12.88% of inertia) vs. second (y axis, 10.15% of inertia) main factors. The different accessions are represented according to the proper epigenotype as defined by the analysis with GenoType and GenoDive.
(TIF)

S4 Fig Factorial Correspondence Analysis based on the results of STRUCTURE analysis. 4A) Factorial Correspondence Analysis showing multivariate relationships among MS-AFLP epigenotypes of different *C*. *sativus* accessions on the axes corresponding to first (x axis, 12.88% of inertia) vs. second (y axis, 10.15% of inertia) main factors. The different groups are represented according to the results of STRUCTURE analysis at K = 2. 4B) Recursive partitioning of population C and D. Population C is structured in seven subpopulations while population D in two subpopulations mainly corresponding to the division Spanish and Non-Spanish samples.
(TIF)

S5 Fig Factorial Correspondence Analysis based on the epigenotypes of the accessions of Spanish origin. Factorial Correspondence Analysis showing multivariate relationships among MS-AFLP epigenotypes of different *C*. *sativus* accessions of Spanish provenance on the axes corresponding to first (x axis, 12.88% of inertia) vs. second (y axis, 10.15% of inertia) main factors. Only the points corresponding to accessions of Spanish origin have been plotted. It is possible to see that eastern (Cuenca and Teruel, blue) and western (Toledo and Ciudad Real, red) clusters are well defined and show only a moderate overlap with each other, while the central cluster (Albacete, green) is in the middle and largely superimposed to both clusters.
(TIF)

S6 Fig Comparison of the epigenetic stability of different saffron crocus accessions in different years. The epigenetic profiles of four accessions (BCU001616, BCU001719, BCU001673, and BCU002476) have been generated using the DNA from leaves sampled in three years (2012, 2013, and 2014). After each sampling, the DNA was extracted and stored at—80°C until the analysis. The methyl sensitive analysis has been carried out by using the enzyme combinations: *Eco*RI/*Hpa*II, as in the present work, and *Eco*RI/*Msp*I. The different profiles (blue—2012, red—2013, and green—2014) are very superimposable and just a few variation have been detected. As an example, the arrows indicate a same DNA fragment obtained with *Eco*RI/*Hpa*II in the different accessions. The fragment is always present in 2012 and 2013 profiles but usually absent in 2014 profiles.
(TIF)

S1 Table List of the saffron crocus accessions used in the present study. (DOCX)

S2 Table List of primers used for AFLPs and MS-AFLPs markers. (DOCX)

S3 Table Indices of clonal diversity for AFLP and MS-AFLP analyses of different saffron crocus accessions. (XLSX)
