# Supplementary figures and images for: AFLP and MS-AFLP Analysis of the Variation within Saffron Crocus (Crocus sativus L.) Germplasm
Source: PLoS One. 2015 Apr 17;10(4):e0123434. doi: 10.1371/journal.pone.0123434 (PMC4401542; doi:10.1371/journal.pone.0123434)

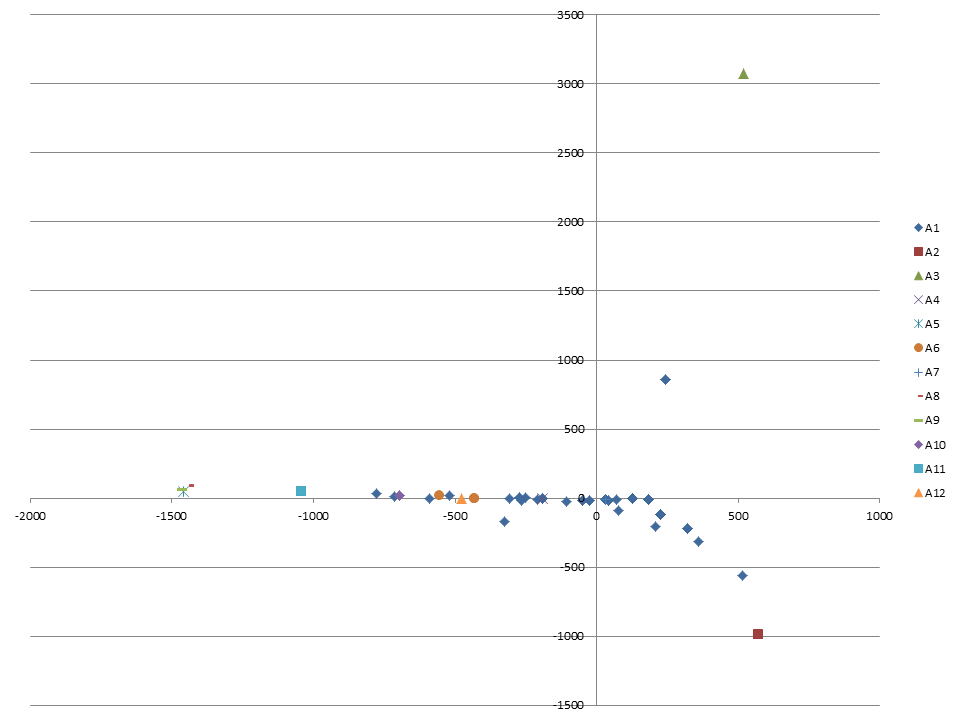

Supplement: S1 Fig — Factorial Correspondence Analysis showing multivariate relationships among AFLP genotypes of different C. sativus accessions on the axes corresponding to first (x axis, 9.72% of inertia) vs. second (y axis, 6.75% of inertia) main factors. The different accessions are represented according to the AFLP genotype, as defined by the analysis with GenoType and GenoDive software. (TIF) [file pone.0123434.s001.tif]

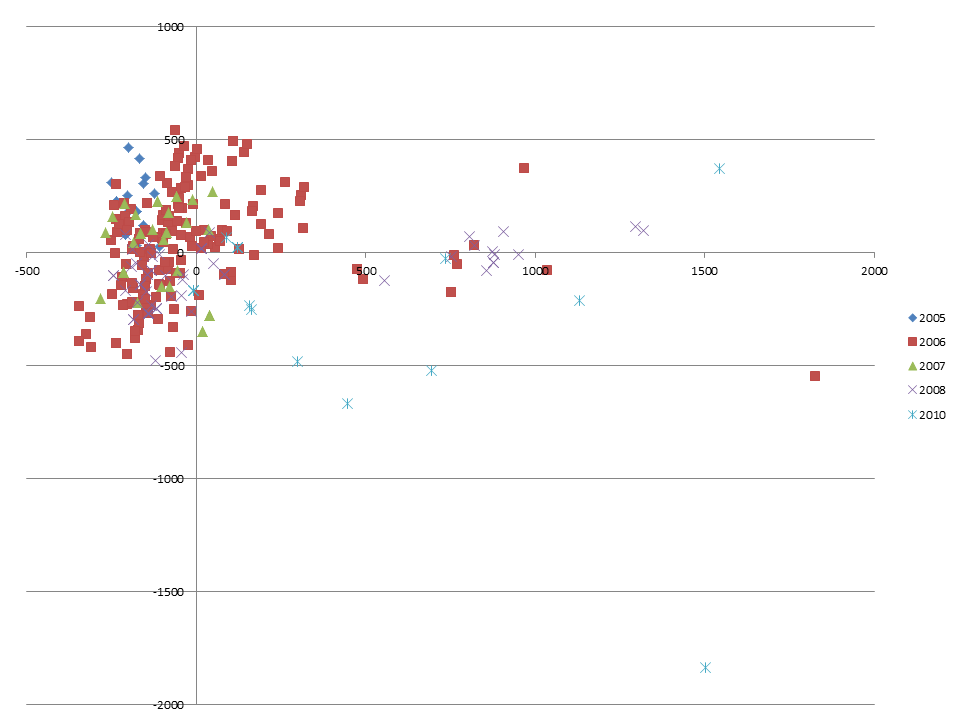

Supplement: S2 Fig — Factorial Correspondence Analysis showing multivariate relationships among MS-AFLP epigenotypes of different C. sativus accessions on the axes corresponding to first (x axis, 12.88% of inertia) vs. second (y axis, 10.15% of inertia) main factors. The accessions are represented according to the year of the first sowing in the germplasm bank. S accessions were received along the entire time while NS accessions were only received in 2006, 2008 and 2010. (TIF) [file pone.0123434.s002.tif]

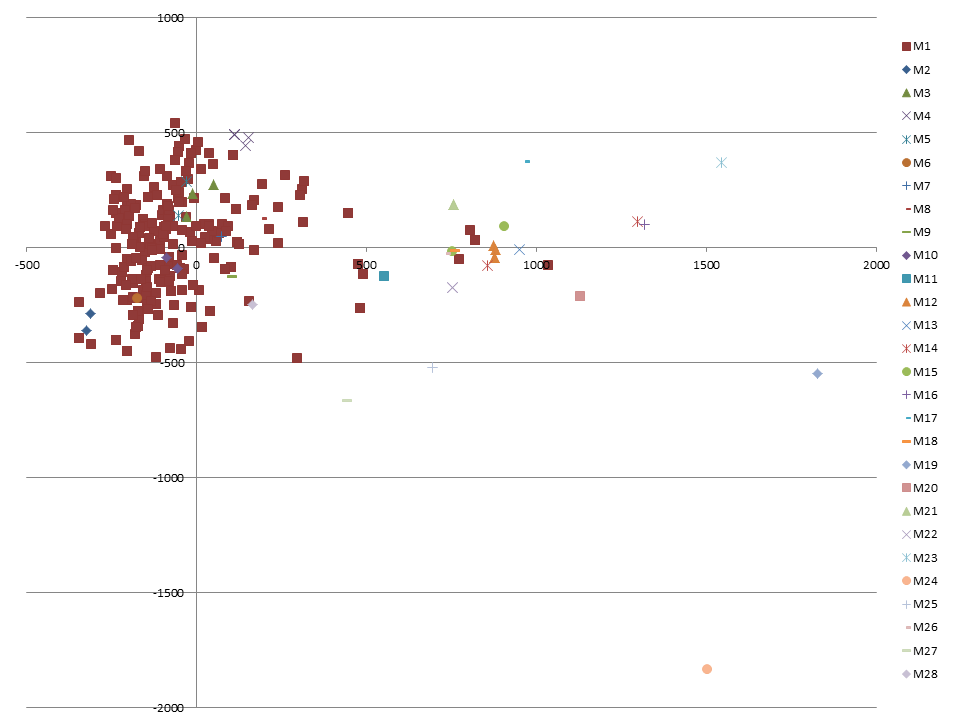

Supplement: S3 Fig — Factorial Correspondence Analysis showing multivariate relationships among MS-AFLP epigenotypes of different C. sativus accessions on the axes corresponding to first (x axis, 12.88% of inertia) vs. second (y axis, 10.15% of inertia) main factors. The different accessions are represented according to the proper epigenotype as defined by the analysis with GenoType and GenoDive. (TIF) [file pone.0123434.s003.tif]

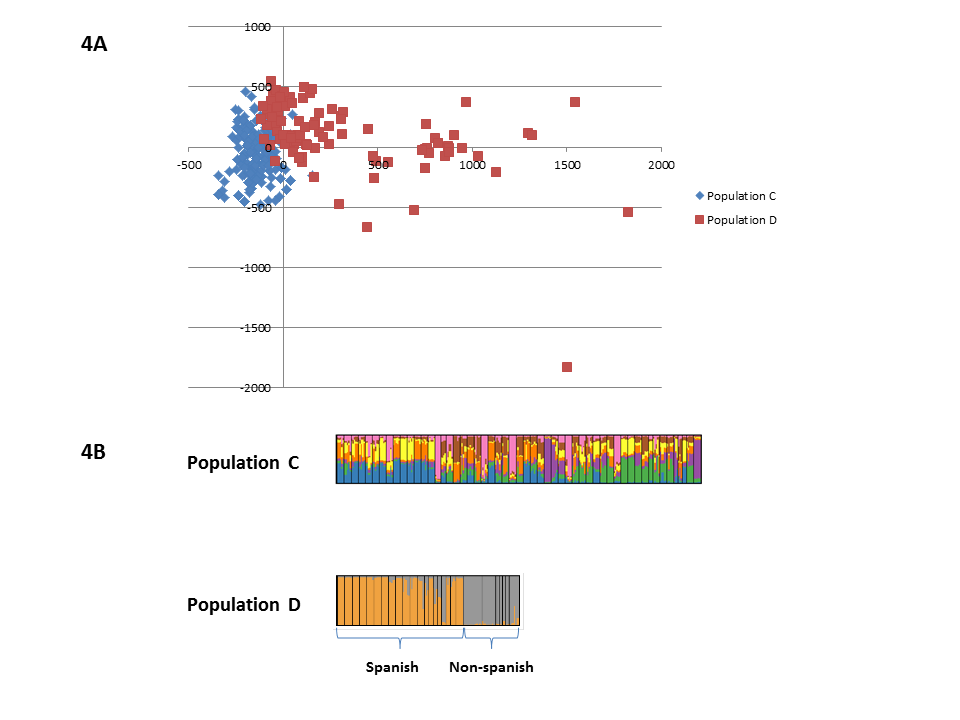

Supplement: S4 Fig — 4A) Factorial Correspondence Analysis showing multivariate relationships among MS-AFLP epigenotypes of different C. sativus accessions on the axes corresponding to first (x axis, 12.88% of inertia) vs. second (y axis, 10.15% of inertia) main factors. The different groups are represented according to the results of STRUCTURE analysis at K = 2. 4B) Recursive partitioning of population C and D. Population C is structured in seven subpopulations while population D in two subpopulations mainly corresponding to the division Spanish and Non-Spanish samples. (TIF) [file pone.0123434.s004.tif]

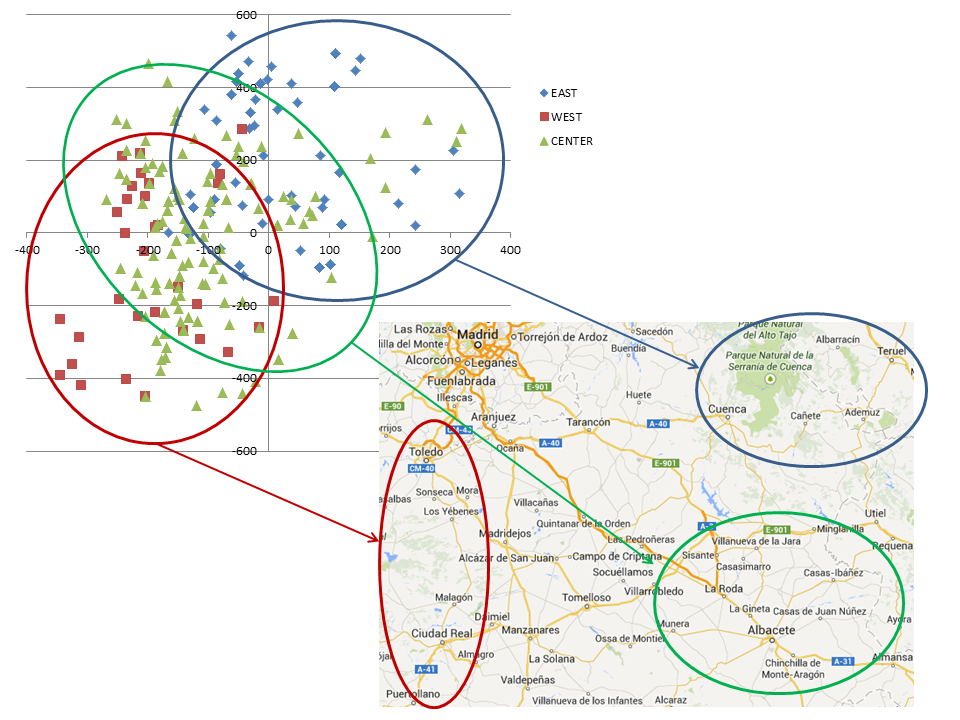

Supplement: S5 Fig — Factorial Correspondence Analysis showing multivariate relationships among MS-AFLP epigenotypes of different C. sativus accessions of Spanish provenance on the axes corresponding to first (x axis, 12.88% of inertia) vs. second (y axis, 10.15% of inertia) main factors. Only the points corresponding to accessions of Spanish origin have been plotted. It is possible to see that eastern (Cuenca and Teruel, blue) and western (Toledo and Ciudad Real, red) clusters are well defined and show only a moderate overlap with each other, while the central cluster (Albacete, green) is in the middle and largely superimposed to both clusters. (TIF) [file pone.0123434.s005.tif]

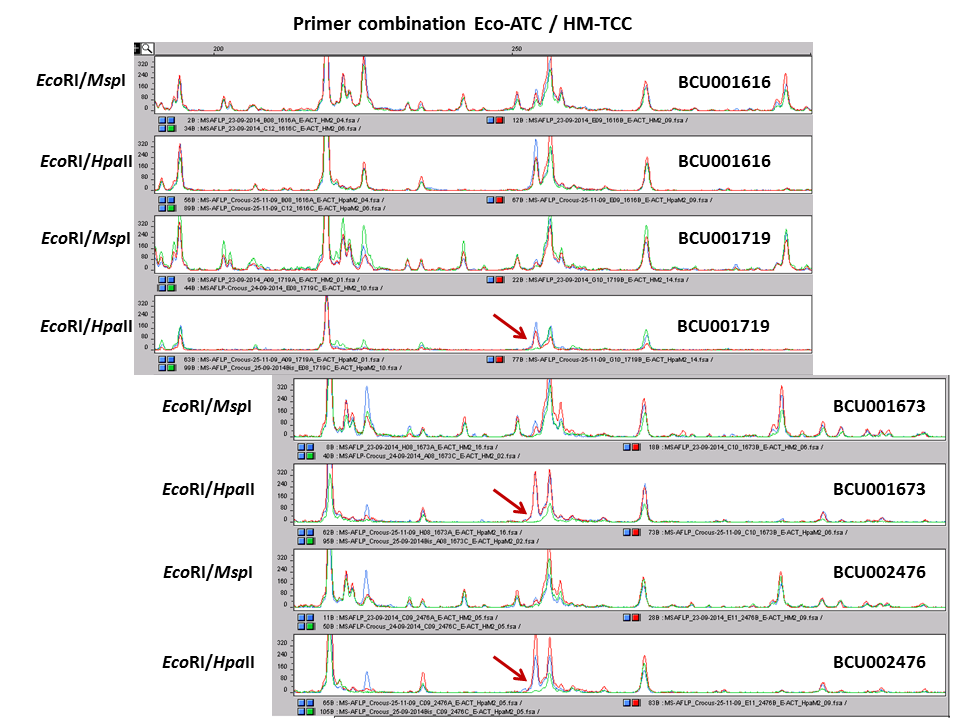

Supplement: S6 Fig — The epigenetic profiles of four accessions (BCU001616, BCU001719, BCU001673, and BCU002476) have been generated using the DNA from leaves sampled in three years (2012, 2013, and 2014). After each sampling, the DNA was extracted and stored at—80°C until the analysis. The methyl sensitive analysis has been carried out by using the enzyme combinations: EcoRI/HpaII, as in the present work, and EcoRI/MspI. The different profiles (blue—2012, red—2013, and green—2014) are very superimposable and just a few variation have been detected. As an example, the arrows indicate a same DNA fragment obtained with EcoRI/HpaII in the different accessions. The fragment is always present in 2012 and 2013 profiles but usually absent in 2014 profiles. (TIF) [file pone.0123434.s006.tif]
