# Supplementary material for: AFLP and MS-AFLP Analysis of the Variation within Saffron Crocus (Crocus sativus L.) Germplasm
Source: PLoS One. 2015 Apr 17;10(4):e0123434. doi: 10.1371/journal.pone.0123434 (PMC4401542; doi:10.1371/journal.pone.0123434)
Supplement: S1 Table — (DOCX) [file pone.0123434.s007.docx]

**Table S1. List of the saffron crocus accessions used in the present study.**

| **A.N.^1^** | **Origin^2^** | **First sowing^3^** | **A.N.** | **Origin** | **First sowing** |
| --- | --- | --- | --- | --- | --- |
| BCU001584 | Spain (A)^4^ | 2005 | BCU001644 | Spain (A) | 2006 |
| BCU001585 | Spain (A) | 2005 | BCU001645 | Spain (A) | 2006 |
| BCU001586 | Spain (A) | 2005 | BCU001646 | Spain (M) | 2006 |
| BCU001587 | Spain (A) | 2005 | BCU001647 | Spain (M) | 2006 |
| BCU001606 | Spain (A) | 2006 | BCU001648 | Spain (A) | 2006 |
| BCU001608 | Spain (A) | 2006 | BCU001668 | Spain (TE) | 2006 |
| BCU001609 | Spain (C) | 2006 | BCU001669 | Spain (TE) | 2006 |
| BCU001610 | Spain (C) | 2006 | BCU001671 | Spain (TE) | 2006 |
| BCU001611 | Spain (A) | 2006 | BCU001672 | Spain (TE) | 2006 |
| BCU001612 | Spain (A) | 2006 | BCU001673 | Spain (TE) | 2006 |
| BCU001613 | Spain (A) | 2006 | BCU001674 | Spain (TE) | 2006 |
| BCU001614 | Spain (CR) | 2006 | BCU001675 | Spain (TE) | 2006 |
| BCU001615 | Spain (T) | 2006 | BCU001677 | Spain (TE) | 2006 |
| BCU001616 | Spain (CR) | 2006 | BCU001678 | Spain (TE) | 2006 |
| BCU001617 | Spain (CR) | 2006 | BCU001679 | Spain (TE) | 2006 |
| BCU001618 | Spain (A) | 2006 | BCU001719 | Spain (C) | 2006 |
| BCU001619 | Spain (T) | 2006 | BCU001722 | Spain (A) | 2007 |
| BCU001620 | Spain (T) | 2006 | BCU001723 | Spain (A) | 2007 |
| BCU001621 | Spain (T) | 2006 | BCU001746 | Spain (I) | 2007 |
| BCU001622 | Spain (T) | 2006 | BCU001747 | Spain (AL) | 2007 |
| BCU001623 | Spain (A) | 2006 | BCU001808 | Spain (A) | 2007 |
| BCU001624 | Spain (CR) | 2006 | BCU001809 | Spain (A) | 2007 |
| BCU001625 | Spain (A) | 2006 | BCU001857 | Spain (M) | 2007 |
| BCU001626 | Spain (A) | 2006 | BCU001858 | Spain (C) | 2007 |
| BCU001627 | Spain (A) | 2006 | BCU002464 | Spain (C) | 2008 |
| BCU001628 | Spain (A) | 2006 | BCU002476 | Spain (LR) | 2008 |
| BCU001630 | Spain (A) | 2006 | BCU002517 | Spain (A) | 2008 |
| BCU001631 | Spain (A) | 2006 | BCU002518 | Spain (A) | 2008 |
| BCU001634 | Spain (A) | 2006 | BCU002819 | Spain (A) | 2008 |
| BCU001636 | Spain (A) | 2006 | BCU002520 | Spain (A) | 2008 |
| BCU001637 | Spain (A) | 2006 | BCU002522 | Spain (A) | 2008 |
| BCU001638 | Spain (A) | 2006 | BCU002523 | Spain (A) | 2008 |
| BCU001639 | Spain (A) | 2006 | BCU002533 | Spain (A) | 2008 |
| BCU001640 | Spain (A) | 2006 | BCU002536 | Spain (A) | 2008 |
| BCU001641 | Spain (A) | 2006 | BCU002865 | Spain (C) | 2010 |
| BCU001642 | Spain (C) | 2006 | BCU002874 | Spain (G) | 2010 |
| BCU001643 | Spain (A) | 2006 | BCU001649 | Morocco (O) | 2006 |

**Table S1** continuation

| **A.N.^1^** | **Origin^2^** | **First sowing^3^** | **A.N.** | **Origin** | **First sowing** |
| --- | --- | --- | --- | --- | --- |
| BCU002477 | France (MP) | 2008 | BCU001693 | Iran (RK) | 2006 |
| BCU002478 | France (MP) | 2008 | BCU001694 | Iran (RK) | 2006 |
| BCU002479 | France (MP) | 2008 | BCU001695 | Iran (SK) | 2006 |
| BCU002480 | France (MP) | 2008 | BCU001690 | Iran (RK) | 2006 |
| BCU002481 | France (MP) | 2008 | BCU001754 | India (S) | 2007 |
| BCU002482 | France (MP) | 2008 | BCU001754 B | India (S) | 2007 |
| BCU002483 | France (MP) | 2008 | BCU001783 | Azerbaijan (B) | 2007 |
| BCU002484 | France (MP) | 2008 | BCU001782 | Italy (CA) | 2007 |
| BCU002485 | France (MP) | 2008 | BCU001806 | Italy (SN) | 2007 |
| BCU002486 | France (MP) | 2008 | BCU001698 | Turkey | 2006 |
| BCU002487 | France (MP) | 2008 | BCU001698 B | Turkey | 2006 |
| BCU002488 | France (MP) | 2008 | BCU001715 | Argentina | 2007 |
| BCU002708 | France (MP) | 2009 | BCU002910 | Greece (K) | 2010 |
| BCU002379 | Afghanistan | 2008 | BCU002911 | Greece (K) | 2010 |
| BCU001687 | Iran (RK) | 2006 | BCU002912 | Greece (K) | 2010 |
| BCU001688 | Iran (RK) | 2006 | BCU002913 | Greece (K) | 2010 |
| BCU001689 | Iran (RK) | 2006 | BCU002914 | Greece (K) | 2010 |
| BCU001691 | Iran (RK) | 2006 | BCU002915 | Greece (K) | 2010 |
| BCU001692 | Iran (RK) | 2006 | BCU002916 | Greece (K) | 2010 |

Germplasm bank number identification, source country and acquisition date of the accessions included in the study.

1) Code of the accession within the BGV-CU; 2) Geographic origin of the samples: A (Albacete, Castilla-La Mancha – Spain), C (Cuenca, Castilla-La Mancha – Spain), CR (Ciudad Real, Castilla-La Mancha – Spain), T (Toledo, Castilla-La Mancha – Spain), G (Guadalajara, Castilla-La Mancha – Spain), M (Murcia, – Spain), TE (Teruel, Aragón – Spain), AL (Alicante, Comunidad Valenciana – Spain), I (Ibiza, Islas Baleares – Spain), LR (La Rioja – Spain), O (Ourzazate – Morocco), MP (Midi-Pyrénées – France), RK (Razavi Khorasan – Iran), SK (South Khorasan – Iran), S (Srinagar, Jammu and Kashmir – India), B (Bilgah, Baku – Azerbaijan), CA (Cagliari, Sardegna – Italy), SN (Siena, Toscana – Italy), K (Khozani – Greece); 3) Acquisition date and year of the first sowing in Cuenca.
