# Supplementary material for: AFLP and MS-AFLP Analysis of the Variation within Saffron Crocus (Crocus sativus L.) Germplasm
Source: PLoS One. 2015 Apr 17;10(4):e0123434. doi: 10.1371/journal.pone.0123434 (PMC4401542; doi:10.1371/journal.pone.0123434)
Supplement: S2 Table — (DOCX) [file pone.0123434.s008.docx]

**S2 Table. List of primers used for AFLPs and MS-AFLPs markers.**

| **Primer** | **Analysis** | **Typology** | **Primer Sequence** |
| --- | --- | --- | --- |
| E01^1^ | AFLP – MS-AFLP | Pre-selective | GACTGCGTACCAATTC**A**^3^ |
| M01^1^ | AFLP | Pre-selective | GATGAGTCCTGAGTAA**A** |
| M02 | AFLP | Pre-selective | GATGAGTCCTGAGTAA**C** |
| HM0^2^ | MS-AFLP | Pre-selective | ATCATGAGTCCTGCTCGG**T** |
| E32 | AFLP – MS-AFLP | Selective | GACTGCGTACCAATTC**AAC** |
| E35 | AFLP | Selective | GACTGCGTACCAATTC**ACA** |
| E36 | AFLP | Selective | GACTGCGTACCAATTC**ACC** |
| E38 | AFLP – MS-AFLP | Selective | GACTGCGTACCAATTC**ACT** |
| E40 | MS-AFLP | Selective | GACTGCGTACCAATTC**AGC** |
| M42 | AFLP | Selective | GATGAGTCCTGAGTAA**AGT** |
| M47 | AFLP | Selective | GATGAGTCCTGAGTAA**CAA** |
| M48 | AFLP | Selective | GATGAGTCCTGAGTAA**CAC** |
| M49 | AFLP | Selective | GATGAGTCCTGAGTAA**CAG** |
| M61 | AFLP | Selective | GATGAGTCCTGAGTAA**CTG** |
| M62 | AFLP | Selective | GATGAGTCCTGAGTAA**CTT** |
| HM1^2^ | MS-AFLP | Selective | ATCATGAGTCCTGCTCGG**TAA** |
| HM2 | MS-AFLP | Selective | ATCATGAGTCCTGCTCGG**TCC** |
| HM3 | MS-AFLP | Selective | ATCATGAGTCCTGCTCGG**TTC** |

1) International code for AFLP primers from the Standard List for AFLP Primer Nomenclature; 2) Custom codes for MS-AFLP MspI pre-selective and selective primers; 3) The selective nucleotides are shown underlined in bold.
